# THE ROLE OF THE ENVIRONMENTAL CONTEXT IN ADVANCE CARE PLANNING AMONG OLDER ADULTS

**DOI:** 10.1093/geroni/igz038.883

**Published:** 2019-11-08

**Authors:** Brittany E Gaines, Kathrin Boerner, Kyungmin Kim, Sara Moorman

**Affiliations:** 1 University of Massachusetts Boston, boston, Massachusetts, United States; 2 Boston College, Chestnut Hill, Massachusetts, United States

## Abstract

Little is known about how environmental context shapes individuals’ advance care planning (ACP). We combined ACP information from the Wisconsin Longitudinal Study with county-level characteristics from the Area Health Resource File, Dartmouth Atlas, and US Census. Multilevel logistic regression models showed that local sociodemographic characteristics (e.g., rurality, racial/ethnic makeup, age composition, and prevalence of one-person households) and healthcare characteristics (e.g., number of hospice agencies, Medicare reimbursement rates) were related to rates of ACP. Additionally, the following environmental factors were moderated by both individual household income and educational attainment, Medicare physician reimbursement rate, racial/ethnic makeup, age composition, median household income, rurality, and the number of hospice agencies. These findings suggest that the environmental context of an individual’s residence can impact their engagement in ACP. Evidence from this study may be used to target areas for, and guide the design of, effective intervention strategies to help increase ACP at an environmental level.

